# Serum free sulfhydryl status associates with new-onset chronic kidney disease in the general population

**DOI:** 10.1016/j.redox.2021.102211

**Published:** 2021-12-09

**Authors:** Arno R. Bourgonje, Amaal E. Abdulle, Martin F. Bourgonje, S. Heleen Binnenmars, Sanne J. Gordijn, Marian L.C. Bulthuis, Sacha la Bastide-van Gemert, Lyanne M. Kieneker, Ron T. Gansevoort, Stephan J.L. Bakker, Douwe J. Mulder, Andreas Pasch, Martin H. de Borst, Harry van Goor

**Affiliations:** a Department of Gastroenterology and Hepatology, University of Groningen, University Medical Center Groningen, Groningen, the Netherlands; b Department of Internal Medicine, Division of Vascular Medicine, University of Groningen, University Medical Center Groningen, Groningen, the Netherlands; c Department of Pathology and Medical Biology, University of Groningen, University Medical Center Groningen, Groningen, the Netherlands; d Department of Internal Medicine, Division of Nephrology, University of Groningen, University Medical Center Groningen, Groningen, the Netherlands; e Department of Obstetrics and Gynecology, University of Groningen, University Medical Center Groningen, Groningen, the Netherlands; f Department of Epidemiology, University of Groningen, University Medical Center Groningen, Groningen, the Netherlands; g Institute for Physiology and Pathophysiology, Johannes Kepler University Linz, Linz, Austria

**Keywords:** Thiols, Sulfur species, Oxidative stress, Chronic kidney disease, Population study, Sulfhydryl groups

## Abstract

**Background:**

Serum sulfhydryl groups (R-SH, free thiols) reliably reflect the systemic redox status in health and disease. As oxidation of R-SH occurs rapidly by reactive oxygen species (ROS), oxidative stress is accompanied by reduced levels of free thiols. Oxidative stress has been implicated in the pathophysiology of chronic kidney disease (CKD), in which redox imbalance may precede the onset of CKD. Therefore, we aimed to investigate associations between serum free thiols and the risk of incident CKD as defined by renal function decline and albuminuria in a population-based cohort study.

**Methods:**

Subjects without CKD (*n* = 4,745) who participated in the Prevention of REnal and Vascular ENd-stage Disease (PREVEND) study, a prospective, population-based cohort study in the Netherlands, were included. Baseline protein-adjusted serum free thiols were studied for their associations with the development of CKD, defined as a composite outcome of an estimated glomerular filtration rate (eGFR) < 60 mL/min/1.73m^2^, urinary 24-h albumin excretion (UAE) > 30 mg/24-h, or both.

**Results:**

Median level of protein-adjusted serum free thiols at baseline was 5.14 μmol/g of protein (interquartile range [IQR]: 4.50–5.75 μmol/g) and median eGFR was 96 mL/min/1.73 m^2^ [IQR: 85–106]. Protein-adjusted serum free thiols were significantly associated with incident CKD (hazard ratio [HR] per doubling 0.42 [95% confidence interval [CI]: 0.36–0.52, *P* < 0.001), even after adjustment for traditional risk factors (HR 0.67 [95% CI: 0.47–0.94], *P*=0.022). In secondary analyses, the highest tertile of protein-adjusted serum free thiols was inversely associated with incident UAE >30 mg/24-h after full adjustment for confounding factors (HR per doubling 0.70 [95% CI: 0.51–0.96], *P*=0.028).

**Conclusion:**

Higher levels of serum R-SH, reflecting less oxidative stress, are associated with a decreased risk of developing CKD in subjects from the general population. This association is primarily driven by incident CKD as defined by UAE.

## Highlights

• Oxidative stress is implicated in the pathophysiology of chronic kidney disease (CKD).
• Serum sulfhydryl groups (R-SH) reflect whole-body redox status in health and disease.
• Serum R-SH associate with new-onset cardiovascular morbidity in many patient cohorts.
• High serum R-SH are associated with a decreased risk of new-onset CKD in the general population.
• Serum R-SH may potentially serve as screening tool for future CKD development.

## Graphical abstract

**Figure:**
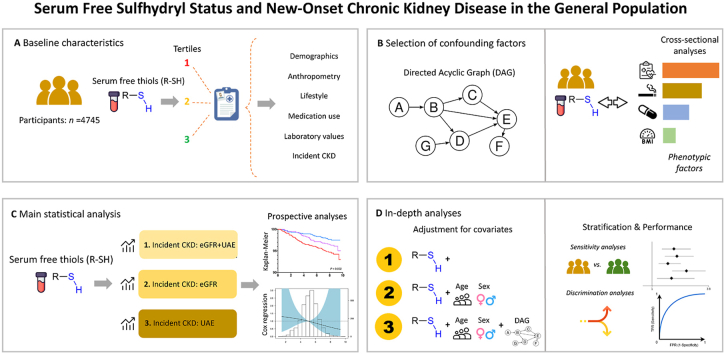

## 1 Introduction

Chronic kidney disease (CKD) affects over 850 million individuals worldwide and is associated with a high risk of cardiovascular morbidity and premature mortality [1]. Early identification and staging of individuals developing CKD is of utmost importance, as early intervention may improve long-term renal health and ultimately prevent or delay the need for dialysis or transplantation [2]. However, the etiology of the disease remains incompletely understood, which also limits the identification of individuals who are at increased risk of developing CKD in the general population [3,4].

Oxidative stress is defined as an imbalance between oxidants and antioxidants in favor of the oxidants, leading to a disruption of redox signaling and control and/or molecular damage [5]. Although reactive species fulfil pivotal physiological functions, overproduction of reactive oxygen species (ROS), may result in oxidative stress which subsequently leads to cellular/molecular damage and tissue destruction [6]. Extracellular free thiols are organosulfur compounds carrying a free sulfhydryl (R-SH) moiety and comprise a robust and reliable reflection of the *in vivo* systemic redox status as they are rapidly oxidized by ROS [7,8]. Some other types of reactive species, however, like hydrogen sulfide (H_2_S) and nitric oxide (NO) can also react with sulfhydryl moieties. Free thiols are the main biological targets of ROS inside and outside of cells; they possess potent antioxidant buffering capacity, and govern a myriad of (protein) functions, enabling both short-term and longer-term biological adaptations [9]. Assessment of serum free thiol concentrations is an easy, minimally invasive and reproducible method of quantifying the degree of systemic oxidative stress [10]. Extracellular free thiol status has been investigated in relation to a variety of (cardiovascular) risk factors (e.g., smoking, alcohol consumption) and is known to be disturbed in a number of oxidative stress-mediated human diseases, e.g. cardiovascular disease, diabetes mellitus, and inflammatory bowel disease [[11], [12], [13], [14]]. Previous studies have also demonstrated that serum free thiols may change upon acute kidney injury (AKI) and that higher levels of serum free thiols are associated with beneficial outcomes and favorable cardiovascular risk profiles in renal transplant recipients [15,16].

As oxidative stress has been acknowledged to play an important role in the pathophysiology of cardiovascular and renal disease [17,18], we hypothesized that free thiols may have merit as a prognostic tool for long-term renal health. However, the value of serum free thiols with regard to the development of CKD is still unclear. In this study, we aimed to investigate whether serum free thiol concentrations are associated with new-onset CKD in subjects derived from the general population.

## 2 Materials and methods

### 2.1 Study population and study design

The Prevention of REnal and Vascular ENd-stage Disease (PREVEND) study is a large-scale, prospective cohort study which was started in 1997 in Groningen, the Netherlands [19]. The PREVEND study was developed to investigate associations between urinary albumin excretion and the occurrence of renal and cardiovascular disease. The study features data on many variables that are relevant to cardiovascular and renal disease from inhabitants of the city of Groningen, aged 28–75 years. In the period of 1997–1998, many inhabitants (*n* = 85,421) were requested to send in a first morning urine sample as well as to complete a short questionnaire on demographics and history of cardiovascular diseases. Subsequently, pregnant women and subjects diagnosed with insulin-treated diabetes mellitus were excluded. A total of 40,856 people (47.8%) responded, of which participants with urinary albumin concentrations (UAC) >10 mg/L (*n* = 7,786) and a randomly selected control group with a UAC <10 mg/L (*n* = 3,395) were invited to participate in subsequent study investigations at the research clinic of the University Medical Center Groningen (UMCG). This screening programme was completed by 8,592 subjects (*n* = 6,000 with UAC >10 mg/L, *n* = 2,592 with UAC <10 mg/L) who eventually formed the full study cohort. A few years later, between 2001 and 2003, a second round of examinations was initiated to collect additional serum samples from 6,894 of these participants. This second visit was considered as the baseline for the present study. From this cohort, patients with CKD (*n* = 1,082) or unknown CKD status (*n* = 337) at time of examination were excluded. Furthermore, participants with missing values on serum free thiols (*n* = 730, due to either missing serum samples or insufficient serum volume) were excluded, which resulted in a final sample size of 4,745 participants for analysis. These excluded participants did demonstrate some statistically significant differences in baseline study population characteristics compared with the included participants (Supplementary Table S1). The study was approved by the Institutional Review Board (IRB) (full name in Dutch: “Medisch Ethische Toetsingscommissie, METc) of the UMCG. All participants provided written informed consent, and the study was conducted in accordance with the principles of the Declaration of Helsinki (2013).

### 2.2 Data collection

Study participants completed a questionnaire asking information about demographics, medical history (e.g. history of cardiovascular disease, renal disease, diabetes), lifestyle habits (e.g., smoking), and medication use. Anthropometric measurements (length, body weight, waist circumference) were performed as well as blood pressure measurements. Blood pressure was automatically measured for 8 min in supine position (Dinamap XL Model 9300 series device, Johnson & Johnson Medical, Tampa, FL). Blood pressure was measured every minute, where the average of the last two measurements was registered as the ultimate blood pressure. Smoking status was noted as “never”, “former” or “current”. Waist circumference was measured on the bare skin at the natural indentation between the 10^th^ rib and the iliac crest.

Fasting blood samples were collected of which aliquots were stored at −80 °C, while urine samples were stored at −20 °C until further analysis. Serum creatinine was measured enzymatically (Roche Modular, Roche Diagnostics, Mannheim, Germany). Serum cystatin C was measured with the Gentian Cystatin C Immunoassay (Gentian AS, Moss, Norway) on a modular analyzer (Roche Diagnostics). Standards were used to calibrate cystatin C according to manufacturer's instructions and following the International Federation of Clinical Chemistry Working Group for Standardization of Serum Cystatin C [20]. High-sensitive C-reactive protein (hs-CRP) was measured using nephelometry (Dade Behring Diagnostics, Marburg, Germany). Participants collected 24-h urine samples for two consecutive days after they were provided with both oral and written instructions. Urinary albumin excretion (UAE) was determined by nephelometry (Dade Behring Diagnostics, Marburg, Germany). UAE was measured twice in two separate 24-h urine collections, and for each participant the average of both was taken for analyses.

### 2.3 Measurement of serum free thiols

Serum free thiols (R-SH, sulfhydryl groups) were measured as described previously, but with minor modifications [21,22]. Serum samples were diluted 4-fold using 0.1 M Tris buffer (pH 8.2). Background absorbance was measured at 412 nm using the Varioskan microplate reader (Thermo Scientific, Breda, the Netherlands), together with a reference measurement at 630 nm. As a next step, 20 μL 1.9 mM 5,5′-dithio-bis(2-nitrobenzoic acid) (DTNB, Ellman's reagent, CAS no. 69-78-3, Sigma-Aldrich Corporation, St. Louis, MO, USA) was added to the samples in 0.1 M phosphate buffer (pH 7.0). Subsequently, samples were incubated for 20 min at room temperature, after which absorbance was measured again. Final serum free thiol concentrations were determined by parallel measurement of an l-cysteine (CAS no. 52-90-4, Fluka Biochemika, Buchs, Switzerland) calibration curve (15.6-1,000 μM) in 0.1 M Tris/10 m EDTA (pH 8.2). Intra- and inter assay coefficients of variation (CV) of serum free thiol measurements were 1.9% and 5.0%, respectively. Finally, serum free thiol concentrations were adjusted to total serum protein (measured according to standard procedures) by calculating the free thiol/total protein ratio (μmol/g of protein). As serum proteins harbor the largest amount of free thiols in the extracellular compartment and therefore largely determine the amount of potentially detectable free thiol groups, this adjustment was performed to indirectly account for this phenomenon and fluid status [9].

### 2.4 Study outcomes and definitions

The primary study outcome was incident CKD which was defined as the first occurrence of either an estimated glomerular filtration rate (eGFR) < 60 mL/min/1.73m^2^, UAE of >30 mg/24-h, or a combination of both. Secondary study outcomes included the first occurrence of an eGFR < 60 mL/min/1.73 m^2^ or UAE > 30 mg/24-h as individual outcomes. Study follow-up ended on January 1, 2011. The eGFR was estimated using the 2012 combined creatinine cystatin C-based Chronic Kidney Disease Epidemiology Collaboration (CKD-EPI) equation [23]. Hypercholesterolemia at baseline was defined by either serum cholesterol levels >6.5 mmol/L, the use of lipid-lowering drugs or serum HDL-cholesterol levels <0.9 mmol/L. Type 2 diabetes mellitus (T2DM) was defined as a fasting glucose level ≥7.0 mmol/L or the use of oral antidiabetics according to the American Diabetes Association (ADA) guidelines.

### 2.5 Statistical analysis

Baseline demographic and clinical characteristics of the study population were presented as means ± standard deviation (SD), medians with interquartile ranges (IQR) in case of skewed variables and as proportions *n* with corresponding percentages (%) in case of nominal variables. Normality was visually assessed using histograms and normal probability (Q-Q) plots. Differences across tertiles of serum free thiol concentrations were tested using one-way analysis of variance (ANOVA) or Kruskal-Wallis tests for continuous variables, while chi-square tests were performed for categorical variables. Univariable and multivariable linear regression analyses were performed to identify variables that were associated with serum free thiol concentrations, while adjusting for relevant covariates derived from univariable analysis (*P*_out_ > 0.05). Standard beta (β) coefficients with corresponding *P*-values were reported indicating strength, direction and statistical significance of the associations. Standardized β-coefficients represented the difference in protein-adjusted serum free thiol levels per 1-SD increment for continuous variables and the difference in protein-adjusted serum free thiols compared to the implied reference group for categorical variables. Linear regression assumptions of residual variance normality and homoscedasticity were verified to confirm absence of violation. Kaplan-Meier survival analysis was performed to assess survival distributions across tertiles of serum free thiol concentrations, which were compared using log-rank tests. Survival time was defined from baseline (second visit, at time of sampling) until the date of the last examination that participants attended, at the incidence of CKD, death, or January 1^st^, 2011 (end of follow-up). Cox proportional hazards regression analyses were performed to assess associations between protein-adjusted serum free thiols and the risk of incident CKD or all-cause mortality, expressed by hazard ratios (HRs) with 95% confidence intervals (CIs). Protein-adjusted serum free thiol concentrations were ^2^log-transformed before entry into Cox regression analyses to facilitate results interpretation (as per doubling). For each predictive factor, the proportionality of hazards assumption was verified to confirm absence of violation. Univariable models were followed by multivariable Cox regression models to adjust for relevant covariates. Stratified analyses were performed to assess HRs across relevant subgroups and to test for potential effect modification by fitting models containing cross-product terms (*P*_interaction_). Additionally, restricted cubic splines (RCS) with three knots were fitted to evaluate potential non-linearity of associations that were established in Cox regression models. Non-linearity was assessed using likelihood ratio tests in which nested models were compared using linear or linear and cubic spline terms. To evaluate the discriminative capacity of the different Cox regression models, receiver operating characteristics (ROC) statistics were computed by calculating the Harrell C-index, which represents the area under the ROC curve (AUC) and is an overall measure of discrimination performance compatible with time-to-event data. Data analysis was performed using R version 3.5.2. (Vienna, Austria) and SPSS Statistics 25.0 (SPSS Inc., Chicago, IL, (USA) and data visualization was performed using R Studio (v.1.2.1335, RStudio, Boston, MA) and the Python programming language (v.3.8.5, Python Software Foundation, https://www.python.org), using the *pandas* (v.1.2.3.), *numpy* (v.1.20.0), *matplotlib* (v.3.4.1) and *seaborn* (v.0.11.1) packages in Python. Two-tailed *P*-values ≤0.05 were considered statistically significant.

### 2.6 Selection of confounding factors: directed acyclic graph (DAG)

Following previously published guidelines on adjustment for confounding factors, causal models (directed acyclic graphs, DAG, and its associated theoretical basis) were used to identify an appropriate set of confounding variables to consider while estimating our outcome of interest [[24], [25], [26]]. The DAG represents the involved causal mechanisms that are hypothesized to underlie the variables at hand (Fig. 1). In this graph, arrows represent the hypothesized causal (direct) effects between variables, whereas the absence of such an arrow represents the assumption of no such a direct effect. Here, we focused on the association between systemic oxidative stress and incident CKD, accompanied by a particular set of confounders for which conditioning was performed in order to produce an unconfounded effect estimate in statistical analyses [[27], [28], [29], [30], [31]]. Based on this framework, the following variables were selected as covariates in the analysis (in addition to age and sex): blood pressure, current smoking, diabetes, history of cardiovascular disease, chronic heart failure, dyslipidemia (defined as the composite of total cholesterol levels and the use of lipid-lowering drugs), and hs-CRP.

**Fig. 1:**
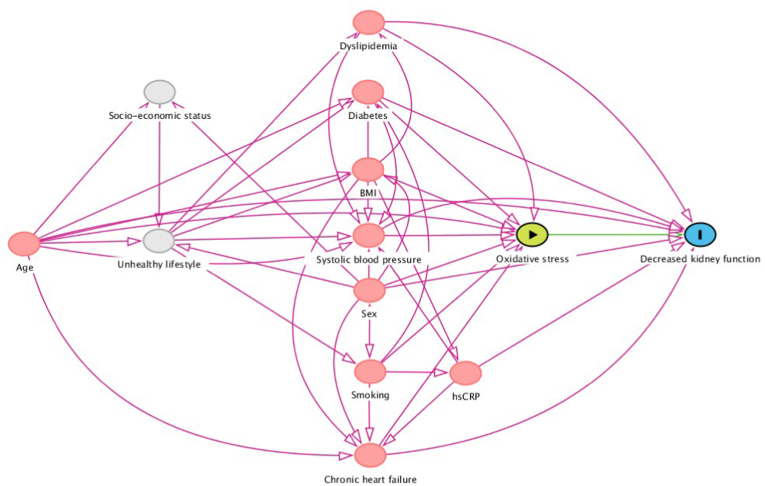
Directed Acyclic Graph (DAG) showing causal relationships hypothesized to underlie the association between systemic oxidative stress (as reflected by serum free thiol levels) and the risk of incident CKD in the general population. Based on the DAG, a specific set of confounding factors was conditioned for in order to obtain an unconfounded effect estimate.

## 3 Results

### 3.1 Baseline cohort characteristics

Baseline characteristics of the study cohort are presented in Table 1, stratified by tertiles of protein-adjusted serum free thiol concentrations (*T1*: <4.75 μmol/g; *T2*: 4.75–5.53 μmol/g; *T3*: >5.53 μmol/g). Mean (±SD) protein-adjusted serum free thiol concentrations were 5.13 (±1.00) μmol/g of protein (median: 5.14 μmol/g of protein, full range: 1.47–8.73 μmol/g). Median age of the study population was 51.4 [IQR: 42.3–58.8] years and 2,551 (53.8%) participants were female. Median eGFR of the study population was 96 [IQR: 85–106] mL/min/1.73m^2^, with higher values among participants within the highest (T3) tertile of protein-adjusted serum free thiols (*P* < 0.001) compared to the remaining participants. Median UAE in the cohort was 7.7 [IQR: 5.8–11.3] mg/24-h. Higher rates of new-onset CKD (all *P* < 0.001) were more often observed in the lowest tertile of protein-adjusted serum free thiol concentrations.

**Table 1:** Baseline demographic and clinical characteristics of the study population, stratified by tertiles of protein-adjusted serum free thiols.

	Total	T1	T2	T3	*P*-value
		4.75 μmol/g	4.75–5.53 μmol/g	5.53 μmol/g	
		*n* = 1581	*n* = 1582	*n* = 1582	
Serum R-SH (protein-adjusted, μmol/g)	5.13 ± 1.00	4.05 ± 0.6	5.14 ± 0.2	6.19 ± 0.6	<0.001
**Demographics**					
Age (years)	51.4 [42.3–58.8]	54.8 [45.7–65.9]	49.8 [42.6–57.8]	46.3 [40.3–53.6]	<0.001
Female, *n* (%)	2551 (53.8)	920 (58.2)	867 (54.8)	764 (48.3)	<0.001
Race, n (%)					0.769
*Caucasian, n (%)*	4517 (95.9)	1512 (96.1)	1505 (95.6)	1500 (96.0)	
*Negroid, n (%)*	45 (1.0)	13 (0.8)	20 (1.3)	12 (0.8)	
*Asian, n (%)*	97 (2.1)	29 (1.8)	34 (2.2)	34 (2.2)	
*Other, n (%)*	52 (1.1)	19 (1.2)	16 (1.0)	17 (1.1)	
**Anthropometrics**					
BMI (kg/m^2^)	25.7 [23.4–28.5]	26.6 [24.1–29.5]	25.8 [23.4–28.4]	25.0 [22.9–27.7]	<0.001
Waist circumference (cm)	90 [81–98]	92 [83–100]	89 [81–98]	88 [80–97]	<0.001
**Risk factors**					
SBP (mmHg)	121 [111–113]	124 [112–137]	120 [111–132]	118 [110–130]	<0.001
DBP (mmHg)	72 [66–78]	72 [67–79]	72 [66–78]	71 [66–77]	0.006
Heart rate (bpm)	68 [62–74]	68 [62–74]	68 [62–74]	67 [61–74]	0.450
Smoking	4686 (98.8)	1564 (98.9)	1563 (98.9)	1559 (98.2)	<0.001
*Never, n (%)*	1466 (31.3)	514 (32.9)	473 (30.3)	479 (30.7)	
*Current, n (%)*	1914 (40.8)	378 (24.2)	429 (27.4)	499 (32.0)	
*Former, n (%)*	1306 (27.9)	672 (43.0)	661 (42.3)	581 (37.3)	
History of CVD, *n* (%)	125 (2.6)	55 (3.5)	39 (2.5)	31 (2.0)	0.025
Diabetes, *n* (%)	71 (1.5)	44 (2.8)	14 (0.9)	13 (0.8)	<0.001
**Medication**					
Antihypertensive drugs, *n* (%)	647 (14.0)	296 (19.3)	190 (12.3)	161 (10.5)	<0.001
Lipid-lowering drugs, *n* (%)	237 (5.1)	108 (7.0)	77 (5.0)	52 (3.4)	<0.001
Antidiabetic drugs, *n* (%)	41 (1.0)	26 (1.9)	6 (0.4)	9 (0.7)	<0.001
**Laboratory measurements**					
Total cholesterol (mmol/L)	5.36 [4.72–6.09]	5.46 [4.85–6.16]	5.35 [4.73–6.13]	5.25 [4.57–6.01]	<0.001
hs-CRP (mg/L)	1.17 [0.56–2.68]	1.43 [0.70–3.37]	1.22 [0.56–2.60]	0.97 [0.46–2.21]	<0.001
eGFR (mL/min/1.73m2)	96 [85–106]	89 [79–101]	96 [86–106]	100 [91–109]	<0.001
UAE (mg/L)	7.7 [5.8–11.3]	7.9 [5.8–12.0]	7.7 [5.8–11.2]	7.6 [5.8–11.0]	0.118
Urine creatinine (mmol/24-h)	11.9 [9.9–14.5]	11.5 [9.7–14.0]	12.0 [10.0–14.5]	12.3 [10.2–14.9]	<0.001
Urine urea (mmol/24-h)	353 [285–427]	350 [284–422]	356 [284–431]	355 [286–426]	0.626
**Study outcomes**					
CKD (eGFR<60 mL/min/1.73m^2^), *n* (%)	155 (3.3)	83 (5.2)	47 (3.0)	25 (1.6)	<0.001
CKD (UAE >30mg/24-h), *n* (%)	363 (7.7)	155 (9.8)	106 (6.7)	102 (6.4)	<0.001
CKD (combination), *n* (%)	482 (10.2)	220 (13.9)	142 (9.0)	120 (7.6)	<0.001

Abbreviations: BMI, body-mass index; CHF, chronic heart failure; CKD, chronic kidney disease; CVD, cardiovascular disease; DBP, diastolic blood pressure; eGFR, estimated glomerular filtration rate; hs-CRP, high-sensitive C-reactive protein; SBP, systolic blood pressure; UAE, urinary albumin excretion.

Univariable and multivariable linear regression analyses were performed to identify independent correlates of protein-adjusted serum free thiols (Table 2). In multivariable analysis, protein-adjusted serum free thiols were higher among current smokers (*β* = 0.068, *P* < 0.001) and positively associated with eGFR (*β* = 0.150, *P* < 0.001), whereas inverse associations were observed with age (*β* = −0.135, *P* < 0.001), female sex (*β* = −0.079, *P* < 0.001), BMI (*β* = −0.065, *P* < 0.001), systolic blood pressure (*β* = −0.075, *P* = 0.001), and hs-CRP (*β* = −0.087, *P* < 0.001).

**Table 2:** Univariable and multivariable linear regression analyses showing associations between protein-adjusted serum free thiols and relevant study parameters.

	St. Beta (β)	*P*-value	St. Beta (β)	*P*-value
	Univariable analysis		Multivariable analysis	
**Demographics**				
Age (years)	−0.269	**<0.001**	−0.135	**<0.001**
Female, *n* (%)	−0.091	**<0.001**	−0.079	**<0.001**
**Anthropometrics**				
BMI (kg/m^2^)	−0.144	**<0.001**	−0.065	**<0.001**
Waist circumference (cm)	−0.110	**<0.001**		
**Risk factors**				
SBP (mmHg)	−0.127	**<0.001**	−0.075	**0.001**
DBP (mmHg)	−0.030	**0.038**		
Heart rate (bpm)	−0.017	0.242		
Current smoking	0.078	**<0.001**	0.068	**<0.001**
History of CVD	−0.039	**0.007**		
Diabetes	−0.070	**<0.001**		
CHF	−0.060	**<0.001**		
**Medication**				
Antihypertensive drugs	−0.019	0.190		
Lipid-lowering drugs	0.012	0.401		
Antidiabetic drugs	−0.056	**<0.001**		
**Laboratory measurements**				
Total cholesterol (mmol/L)	−0.064	**<0.001**		
hsCRP (mg/L)	−0.132	**<0.001**	−0.087	**<0.001**
eGFR (mL/min/1.73m^2^)	0.270	**<0.001**	0.150	**<0.001**
UAE (mg/L)	−0.028	0.055		
Urine creatinine (mmol/24-h)	0.089	**<0.001**		
Urine urea (mmol/24-h)	0.011	0.464		

Abbreviations: BMI, body-mass index; CHF, chronic heart failure; CVD, cardiovascular disease; DBP, diastolic blood pressure; eGFR, estimated glomerular filtration rate; hs-CRP, high-sensitive C-reactive protein; SBP, systolic blood pressure; UAE, urinary albumin excretion.

### 3.2 Protein-adjusted serum free thiols and incident CKD

**Fig. 2:**
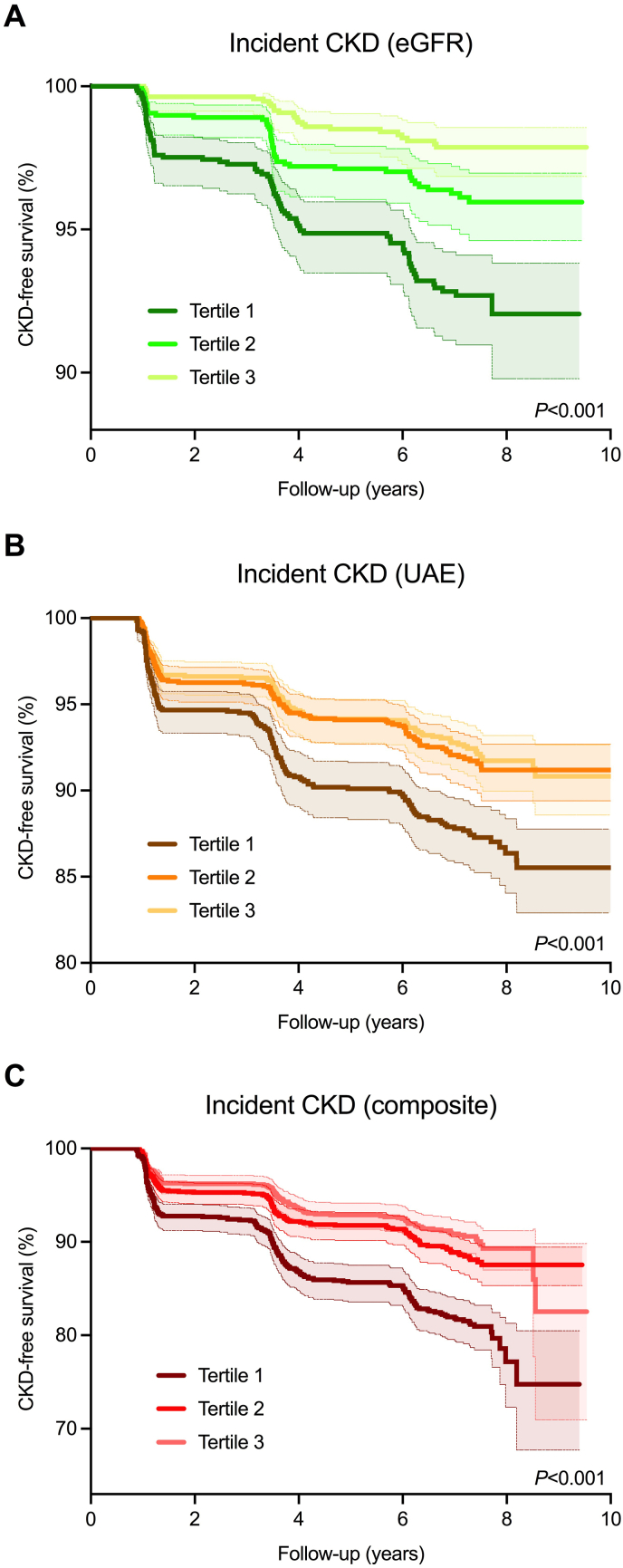
(A–C). Kaplan-Meier survival curves for tertiles of protein-adjusted serum free thiols, representing CKD-free survival based on the individual determinants of eGFR (<60 mL/min/1.73 m^2^) and UAE (>30 mg/24-h) as well as on the composite outcome of incident CKD (eGFR, UAE or both). Highest rates of incident CKD were observed in the lowest tertile (T1) of protein-adjusted serum free thiols (log-rank tests, all P < 0.001).

**Fig. 3:**
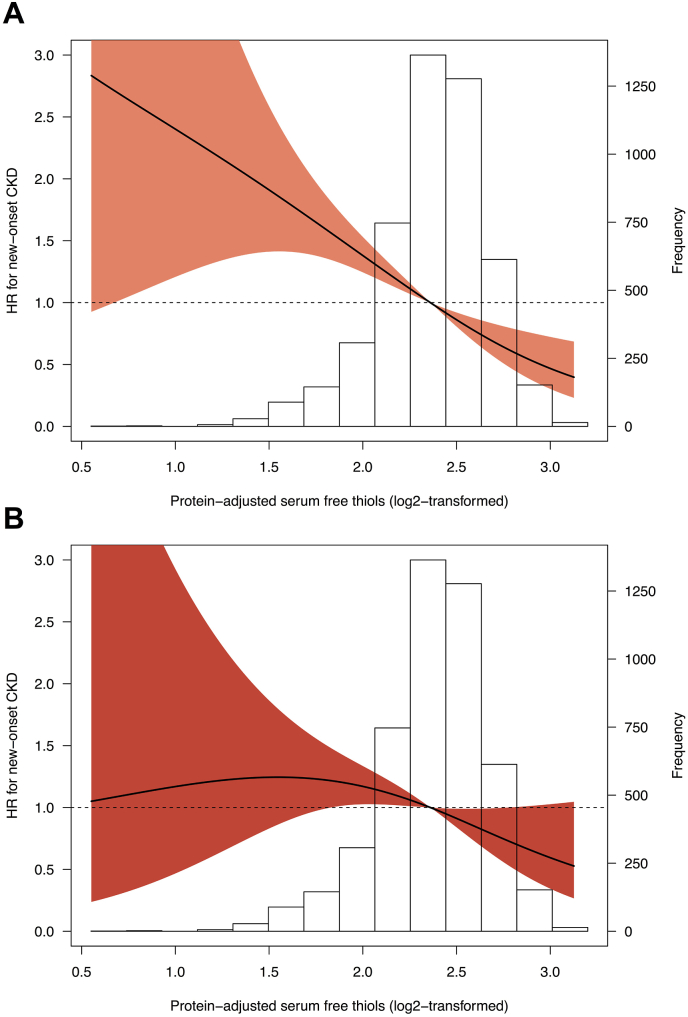
(A–B). Restricted cubic splines (RCS) demonstrating the association between protein-adjusted serum free thiols and the risk of incident CKD for both (A) the crude model (Model 1) and (B) the fully adjusted model (Model 4). Estimated associations were derived from Cox proportional hazards regression models and RCS based on three knots (0.5th, 50th and 99.5th percentiles). Likelihood ratio tests for non-linearity were not significant for both models (χ^2^ =1.32, P=0.287 and χ^2^ =1.11, P=0.292, respectively). Orange- and red-shaded areas represent 95% confidence intervals. (For interpretation of the references to colour in this figure legend, the reader is referred to the Web version of this article.)

During a median follow-up of 8.4 [IQR: 7.9–8.9] years, 482 participants had incident CKD, of which 155 participants based on eGFR (<60 mL/min/1.73m^2^), 363 based on UAE (>30 mg/24-h) and 36 participants developed both. The highest rate of incident CKD occurred in the lowest tertile (T1) of protein-adjusted serum free thiols (*n* = 251, 15.9%, *P* < 0.001). Kaplan-Meier survival analysis demonstrated statistically significant differences in survival distributions among tertiles of protein-adjusted serum free thiols for incident CKD, both for the composite outcome as well as for individual outcomes based on either eGFR or UAE (Fig. 2, log-rank tests, all *P* < 0.001). Cox proportional hazards regression analyses demonstrated a statistically significant association with the risk of incident CKD (Table 3A, *Model 1*, hazard ratio [HR] per doubling of protein-adjusted serum free thiols 0.42 [95% CI: 0.36–0.52], *P* < 0.001). After adjustment for the identified confounding factors (age, sex, systolic blood pressure, total cholesterol, use of lipid-lowering drugs, diabetes, current smoking, chronic heart failure, hs-CRP), this association remained statistically significant (Table 3A, *Model 4*, HR per doubling 0.67 [95% CI: 0.47–0.94], *P*=0.022). Discriminative capacity of these Cox proportional hazards regression models demonstrated significant improvement after adjustment for potential confounding factors (Harrell C indices 0.58 and 0.73, respectively, Supplementary Fig. S1). Restricted cubic splines (RCS) showed no significant deviations from linear associations with the risk of incident CKD (Fig. 3, *P*=0.287 and *P*=0.292 for the crude and fully adjusted models, respectively). Subsequently, when analyzing prospective associations between protein-adjusted serum free thiols and the individual components of the composite incident CKD outcome (i.e. eGFR <60 mL/min/1.73m^2^ and UAE >30 mg/24-h), similar associations were observed (eGFR: Table 3B, *Model 1*, HR per doubling 0.26 [95% CI: 0.17–0.40], *P* < 0.001; UAE: Table 3C, *Model 1*, HR per doubling 0.53 [95% CI: 0.38–0.72], *P* < 0.001). However, statistical significance vanished after adjustment for the identified confounding factors when protein-adjusted serum free thiols was examined as continuous variable. Still, the highest tertile (T3) of protein-adjusted serum free thiols was significantly associated with incident UAE >30 mg/24-h after full adjustment for confounding factors (Table 3C, *Model 4*, HR per doubling 0.70 [95% CI: 0.51–0.96], *P*=0.028).

**Table 3:** Cox proportional hazards regression analyses for associations between protein-adjusted serum free thiols and the risk of incident CKD, either as composite outcome or based on its individual determinants (eGFR and UAE).

	HR per doubling	T1	T2	T3
		<4.75 μmol/g	4.75–5.53 μmol/g	>5.53 μmol/g
**A. CKD (composite outcome) (*n*=482)**				
Model 1	0.42 [0.32–0.56], ***P*<0.001**	1.00 (reference)	0.58 [0.47–0.72], ***P*<0.001**	0.49 [0.39–0.61], ***P*<0.001**
Model 2	0.66 [0.49–0.89], ***P*=0.007**	1.00 (reference)	0.72 [0.58–0.89], ***P*=0.002**	0.72 [0.57–0.90], ***P*=0.005**
Model 3	0.68 [0.51–0.93], ***P*=0.015**	1.00 (reference)	0.73 [0.59–0.91], ***P*=0.005**	0.74 [0.58–0.93], ***P*=0.011**
Model 4	0.67 [0.47–0.94], ***P*=0.022**	1.00 (reference)	0.77 [0.60–0.98], ***P*=0.032**	0.67 [0.51–0.88], ***P*=0.005**
**B. CKD (eGFR<60 mL/min/1.73m**^**2**^**) (*n*=155)**				
Model 1	0.26 [0.17–0.40], ***P*<0.001**	1.00 (reference)	0.52 [0.37–0.75], ***P*<0.001**	0.28 [0.18–0.43], ***P*<0.001**
Model 2	0.27 [0.44–1.26], *P*=0.268	1.00 (reference)	0.91 [0.63–1.31], *P*=0.910	0.71 [0.45–1.13], *P*=0.712
Model 3	0.79 [0.46–1.34], *P*=0.374	1.00 (reference)	0.94 [0.65–1.36], *P*=0.725	0.74 [0.47–1.18], *P*=0.204
Model 4	0.75 [0.42–1.34], *P*=0.331	1.00 (reference)	0.95 [0.65–1.41], *P*=0.808	0.60 [0.35–1.01], *P*=0.056
**C. CKD (UAE >30mg/24-h) (*n*=363)**				
Model 1	0.53 [0.38–0.72], ***P*<0.001**	1.00 (reference)	0.63 [0.50–0.81], ***P*<0.001**	0.60 [0.47–0.77], ***P*<0.001**
Model 2	0.68 [0.48–0.95], ***P*=0.025**	1.00 (reference)	0.71 [0.56–0.91], ***P*=0.008**	0.74 [0.57–0.96], ***P*=0.024**
Model 3	0.71 [0.50–1.00], *P*=0.052	1.00 (reference)	0.73 [0.57–0.94], ***P*=0.015**	0.76 [0.58–0.99], ***P*=0.043**
Model 4	0.69 [0.46–1.04], *P*=0.073	1.00 (reference)	0.77 [0.58–1.03], *P*=0.077	0.70 [0.51–0.96], ***P*=0.028**

Model 1, crude model. Model 2, model 1 with adjustment for age and sex. Model 3, model 2 with adjustment for current smoking, systolic blood pressure, chronic heart failure, diabetes, total cholesterol, use of lipid-lowering drugs. Model 4, model 3 with adjustment for high-sensitive C-reactive protein (hs-CRP). Bold *P*-values indicate statistical significance. Abbreviations: HR, hazard ratio; CKD, chronic kidney disease; eGFR, estimated glomerular filtration rate; UAE, urinary albumin excretion.

### 3.3 Stratified analyses

**Fig. 4:**
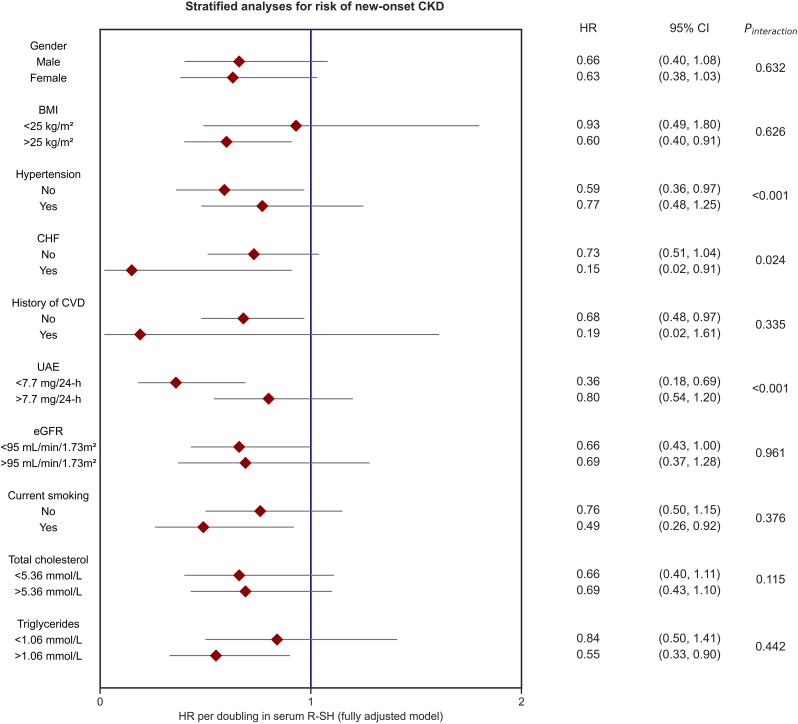
Stratified analyses for the association between protein-adjusted serum free thiols and the risk of incident CKD across various subgroups. Hazard ratios (HRs) are shown with corresponding 95% confidence intervals (CIs). HRs show consistently inverse associations between protein-adjusted serum free thiols and the risk of incident CKD in all subgroups. HRs were adjusted for potential confounding factors (based on the DAG), including current smoking, systolic blood pressure, chronic heart failure, diabetes, total cholesterol, use of lipid-lowering drugs, and hs-CRP. Abbreviations: BMI, body-mass index; CHF, chronic heart failure; CKD, chronic kidney disease; CVD, cardiovascular disease; eGFR, estimated glomerular filtration rate; R-SH, protein-adjusted serum free thiols; UAE, urinary albumin excretion; HR, hazard ratio; CI, confidence interval.

Stratified analyses for the association between protein-adjusted serum free thiols and the risk of incident CKD demonstrated consistently inverse associations in all analyzed subgroups (Fig. 4, Supplementary Table S2). Notably, stratification by the presence of hypertension, chronic heart failure, and by median UAE showed significant effect modification (*P*_interaction_ <0.001, 0.024 and <0.001, respectively). Corresponding HRs were lower for participants without hypertension and with below-median UAE, whereas HRs were higher among participants without chronic heart failure.

### 3.4 Sensitivity analyses

Results of the fully adjusted model for the association between protein-adjusted serum free thiols and the risk of incident CKD did not substantially change when subjects with an UAE >25 mg/24-h (instead of 30 mg/24-h) at baseline were excluded from the analyses, in which ^2^log-transformed protein-adjusted serum free thiols remained significantly associated with incident CKD (HR 0.66 [95% CI: 0.45–0.96], *P*=0.029, respectively). Similarly, when subjects with eGFR <65 mL/min/1.73 m^2^ (instead of <60 mL/min/1.73 m^2^) at baseline were excluded, the association between protein-adjusted serum free thiols and new-onset CKD remained similar, although lost statistical significance (HR 0.75 [95% CI: 0.51–1.11], *P*=0.152). Furthermore, when we excluded subjects who used antihypertensive drugs at baseline, we observed the association between protein-adjusted serum free thiols and incident CKD to become even stronger (HR 0.55 [95% CI: 0.34–0.83], *P*=0.006, fully adjusted model).

## 4 Discussion

Protein-adjusted serum free thiols, as surrogate measure of systemic oxidative stress, are significantly associated with the risk of incident CKD in the general population within a follow-up of almost 10 years. After adjustment for traditional risk factors for CKD, the association between protein-adjusted serum free thiols and incident CKD remained statistically significant. Although individual determinants defining incident CKD (either <60 mL/min/1.73m^2^ or an UAE >30 mg/24-h) were not significantly associated to baseline protein-adjusted serum free thiols (as continuous parameter) when adjusting for confounders, the highest tertile of serum free thiols remained significantly inversely associated to incident UAE (defined as >30 mg/24-h). In addition, stratified analyses across various subgroups indicated consistently inverse associations between protein-adjusted serum free thiols and incident CKD. Collectively, these results highlight the potential of extracellular free thiols as a risk stratification tool in preventive settings in order to attenuate future CKD burden in the general population.

Although the underlying etiology of CKD is complex, oxidative stress has previously been found to play an important role in the development of renal impairment [17]. The endothelium, which is an essential component in the regulation of physiological renal function, is highly susceptible to oxidative damage [32]. Moreover, there is increasing evidence that oxidative stress results in autophagy and apoptosis in podocytes and mesangial cells, promotes activation of profibrotic pathways and glomerulosclerosis, and directly influences the permeability of the glomerular basement membrane, thereby altering glomerular filtration [33]. In addition, oxidative stress is also believed to be associated with complications (e.g., hypertension, atherosclerosis, inflammation) in patients with advanced CKD, and with the acceleration of kidney disease progression [34]. However, the potential role of oxidative stress in association with renal function impairment in the general population has not been clearly established. The current study is the first to clearly demonstrate the association between free thiols and kidney function decline in the general population.

The current study included 482 (10.2%) participants who developed incident CKD within a follow-up of almost 10 years, with the highest rate of incident CKD found in participants within the lowest tertile (T1) of protein-adjusted serum free thiols (T1: 13.9% vs. T2: 9.0% and T3: 7.6%). This finding is in line with previous studies that have shown a significantly increased level of oxidative stress even in patients who were in the early stages of CKD [35,36]. Moreover, we demonstrated a significant inverse association with the risk of incident CKD per doubling of protein-adjusted serum free thiols. This association remained statistically significant after adjustment for potential confounding factors (including age, sex, systolic blood pressure, total cholesterol, use of lipid-lowering drugs, diabetes, current smoking, chronic heart failure, hs-CRP). However, statistical significance of this multivariable model disappeared when additional adjustment for baseline eGFR was performed. Considering these observations, we hypothesize that the decline of renal function is preceded by endothelial dysfunction. Oxidative stress is known to damage the endothelial glycocalyx, a critical barrier on the surface of endothelial cells known to be involved in renal disease [37]. Damage to the glycocalyx causes mitochondrial deterioration with subsequent mitochondrial ROS production, thereby inducing a vicious circle of damage. In line with our findings, it was previously reported that plasma malondialdehyde (MDA), a marker of ROS-induced lipid peroxidation, is significantly associated with the prevalence of mild insufficiency of kidney function and chronic kidney disease [38].

Serum free thiols (R-SH, sulfhydryl groups) are crucial components of the extracellular antioxidant machinery and reliably reflect the systemic *in vivo* redox status, as they are able to readily scavenge ROS, while they also act as multimodal redox relays, kinetically controlling intra- and extracellular redox exchange reactions [39]. Extracellular free thiol groups mainly comprise circulating cysteine-based proteins, of which albumin is the most relevant example - not only because of its redox properties, but also because it is one of the most abundant plasma proteins [7,40]. In addition, free thiols occur in low-molecular-weight (LMW) compounds, e.g. glutathione and homocysteine. Collectively, the sum of low- and high-molecular weight thiols are referred to as *total free thiols,* which have a major impact on the net extracellular redox equilibrium [41]. Higher levels of extracellular free thiols indicate a more favorable redox status and associate with many health parameters, whereas reduced extracellular free thiol levels arise from oxidation due to ROS overproduction, which, in turn, is associated with various cardiovascular risk factors, including ageing, obesity, alcohol consumption and hypertension [9,12]. Apart from rather statically reflecting the global extracellular redox status in health and disease conditions, extracellular free thiols may also be considered rather dynamic biomarkers. Regardless of the potential factors that are responsible for dynamic changes in extracellular free thiols, rapid changes in their levels may be the result of a balanced act between oxidizing and reducing events targeting the single free cysteine thiol (Cys^34^) of albumin, the latter compound making the greatest contribution to extracellular free thiol status [7]. For instance, a previous study observed a rapid increase in extracellular free thiols following transient hypothermia-induced vasoconstriction in patients with primary Raynaud's phenomenon and systemic sclerosis [42]. Another example constitutes a study in which extracellular free thiols were observed to change dramatically over the course of several days in patients having critical illness [43]. These observations underscore the reversibility and dynamic nature of processes that are related to oxidative modification of extracellular free thiols, thereby lowering their total availability in the systemic circulation. As the availability of circulating proteins largely determines the amount of potentially detectable extracellular free thiol groups, an adjustment to total protein (or albumin) may be considered as an indirect approach to more accurately reflect the total extracellular free thiol pool [12,16,42,44,45]. A single, easily reproducible, robust, minimally invasive quantification of serum free thiols may become a useful screening tool for measuring whole-body redox status in a variety of clinical contexts.

In stratified analyses, an important observation was that protein-adjusted serum free thiols and the risk of incident CKD remained consistently inversely associated in all analyzed subgroups, although few of them without statistical significance. Notably, associations between serum free thiols and the risk of incident CKD showed significantly stronger associations in subgroups characterized by below-median UAE, the absence of hypertension, and the presence of chronic heart failure. A possible explanation for the latter result could be that chronic heart failure strengthens the prediction as this condition is already by itself accompanied by oxidative stress [11]. Furthermore, CHF is strongly associated with renal dysfunction, not only because of the use of medical therapies affecting renal haemodynamics and solute handling (diuretics), but also because the mutual interactions between the heart and the kidney are critical for proper execution of their physiological functions [46]. However, these findings should be interpreted with caution as several subgroup sizes were rather imbalanced, which may have introduced bias.

Currently, serum creatinine remains the primary index for the detection and monitoring of the prevalence and severity of kidney disease [47]. More recently the KDIGO (Kidney Disease: Improving Global outcomes) classification was enriched with the addition of urinary albumin to creatinine ratio (UACR) meant for CKD staging and prognostication [1]. By doing so, CKD measures include both estimated kidney function (eGFR) as well as the endothelial injury-reflecting UACR. Additional markers related to damage of the tubular compartment such as kidney injury molecule-1 (KIM-1) and monocyte chemotactic protein-1 (MCP-1) have been investigated intensively and also add to the diagnosis of CKD [48,49]. Oxidative stress is an early event in hypoxic and inflammatory conditions and relates to various comorbidities of CKD including high BMI and vascular damage. In the present study, we convincingly show that a robust biomarker of oxidative stress is strongly associated with incident CKD in the general population. This emphasizes that markers of oxidative stress may aid in predicting the occurrence of CKD.

Aside from the potential prognostic value of protein-adjusted serum free thiols, our results also highlight opportunities for future development of therapeutic and preventive interventions. As free thiols may be receptive to therapeutic modulation, exogenous administration of thiol-targeted agents could be a potential strategy as these compounds may restore extracellular thiol homeostasis in circumstances of pathological overproduction of ROS [[50], [51], [52]]. However, following this therapeutic approach, thiol-based antioxidants should ideally not disturb the physiological functions of ROS, but rather stay reserved for individuals who have evident free radical overproduction, which may be reflected by reduced levels of serum free thiols. Extracellular free thiols may be considered as an integrative marker of whole-body redox status and may serve as an important tool for interrogating an individual's redox phenotype and aid in risk stratification in primary and secondary prevention settings in the context of CKD. In this context, patient stratification based on redox phenotyping would inform future therapeutic decision-making. Importantly, treatment decisions should be tailored to the specific level of “redox stresses”, selecting a personalized intervention to achieve whole-body redox balance [53]. This, in turn, could improve the effectiveness of such therapies, as it may lower the risk of eliciting further perturbances to redox signaling systems by incautious administration of thiol-based antioxidants, instead of protecting them from the potential negative effects of additional stress events. In light of these considerations, the question whether incident CKD could be prevented by modification of the extracellular free thiol content remains to be answered in future studies.

Strengths of the present study include the size and extensive phenotypic characterization of the study cohort as well as the relatively long follow-up duration, which enabled us to prospectively evaluate the prognostic value of serum free thiols with regard to the development of CKD in a population-based setting, while controlling for relevant covariates in the analysis. At the same time, several limitations of this study warrant recognition. For instance, the observational nature of the study limits causal inference for the relationship between free thiols and incident CKD. Another limitation constitutes the poor generalizability of our results to other ethnicities and geographical locations, as most participants in this study were Caucasians who lived in the northern parts of the Netherlands, respectively. In addition, we were forced to exclude a number of participants from the present study of whom no serum samples or insufficient serum volume was available to determine protein-adjusted serum free thiol concentrations. This group of participants demonstrated statistically significant differences in baseline study population characteristics, which might have introduced exclusion bias, which could, however, not be completely avoided.

## 5 Conclusions

Taken together, we conclude that serum free thiols are significantly associated with the risk of incident CKD in the general population. Serum free thiols might harbor considerable potential as an easily measurable biomarker for monitoring the risk of future renal function decline. Future prospective studies are warranted to further elucidate the clinical relevance of extracellular free thiols, which is also of potential significance as these compounds may be amenable to therapeutic intervention.

## Funding

The Prevention of REnal and Vascular ENd-stage Disease (PREVEND) study was supported by grants from the 10.13039/501100002997Dutch Kidney Foundation (grant no. E.013).

## Author contributions

ARB, AEA, RTG, SJLB, AP, MHdB and HvG were involved in conceptualization and study design. RTG, SJLB, AP and HvG were responsible for funding acquisition and resources. RTG, SJLB, AP and HvG collected all study data. ARB performed data analysis and data visualization. ARB, AEA, MFB and HvG wrote the first draft of the manuscript. All authors contributed to results interpretation, critically reviewed the manuscript, contributed to manuscript revision, and read and approved the final version of the manuscript to be submitted for publication.

## Ethical considerations

This study was reviewed and approved by the Institutional Review Board (IRB) of the University Medical Center Groningen (UMCG), Groningen, the Netherlands (Dutch: “Medisch Ethische Toetsingscommissie,” METc). All participants provided written informed consent for their participation in the study. The study was conducted in accordance with the principles of the Declaration of Helsinki.

## Data availability statement

The datasets generated for this study are available on reasonable request to the corresponding author.

## Declaration of competing interest

The authors have no conflicts of interest to declare.

## Figures and Tables

**figs1:**
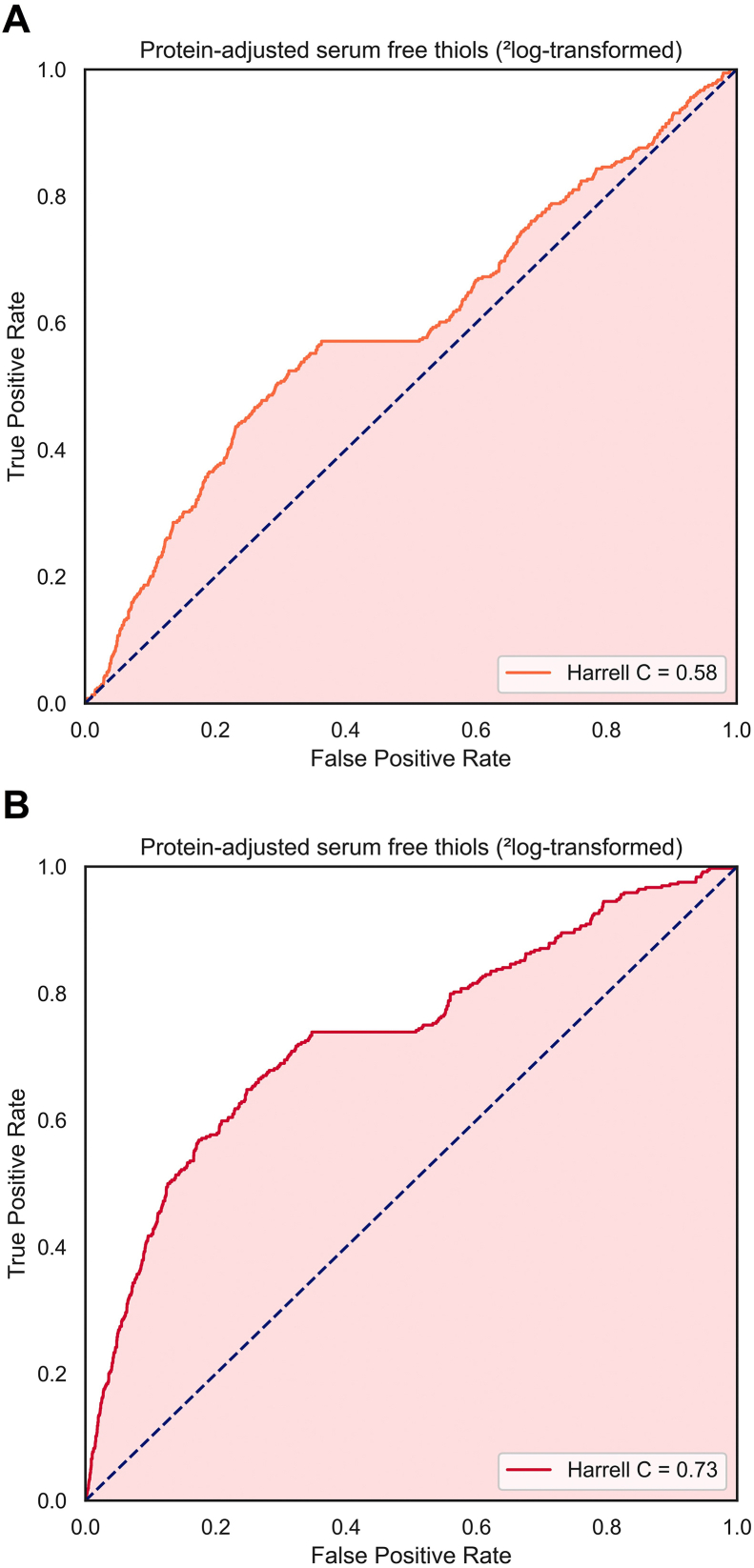


## References

[1] (2013). KDIGO 2012 clinical practice guideline for the evaluation and management of chronic kidney disease. Kidney Disease: Improving Global Outcomes CKD Work Group. Kidney Int. Suppl..

[2] Gansevoort R.T., de Jong P.E. (2009). The case for using albuminuria in staging chronic kidney disease. J. Am. Soc. Nephrol..

[3] Shlipak M.G., Day E.C. (2013). Biomarkers for incident CKD: a new framework for interpreting the literature. Nat. Rev. Nephrol..

[4] De Jong M.A., Eisenga M.F., van Ballegooijen A.J., Beulens J.W.J., Vervloet M.G., Navis G. (2021). Nephrol. Dial. Transplant..

[5] Sies H. (2015). Oxidative stress: a concept in redox biology and medicine. Redox Biol.

[6] Sies H., Jones D.P. (2020). Reactive oxygen species (ROS) as pleiotropic physiological signalling agents. Nat. Rev. Mol. Cell Biol..

[7] Turell L., Radi R., Alvarez B. (2013). The thiol pool in human plasma: the central contribution of albumin to redox processes. Free Radic. Biol. Med..

[8] Banne A.F., Amiri A., Pero R.W. (2003). Reduced level of serum thiols in patients with a diagnosis of active disease. J. Anti Aging Med..

[9] Cortese-Krott M.M., Koning A., Kuhnle G.G.C., Nagy P., Bianco C.L., Pasch A. (2017). The reactive species interactome: evolutionary emergence, biological significance, and opportunities for redox metabolomics and personalized medicine. Antioxidants Redox Signal..

[10] Bourgonje A.R., Feelisch M., Faber K.N., Pasch A., Dijkstra G., van Goor H. (2020). Oxidative stress and redox-modulating therapeutics in inflammatory bowel disease. Trends Mol. Med..

[11] Koning A.M., Meijers W.C., Pasch A., Leuvenink H.G.D., Frenay A.S., Dekker M.M. (2016). Serum free thiols in chronic heart failure. Pharmacol. Res..

[12] Abdulle A.E., Bourgonje A.R., Kieneker L.M., Koning A.M., la Bastide-van Gemert S., Bulthuis M.L.C. (2020). Serum free thiols predict cardiovascular events and all-cause mortality in the general population: a prospective cohort study. BMC Med..

[13] Schillern E.E.M., Pasch A., Feelisch M., Waanders F., Hendriks S.H., Mencke R. (2019). Serum free thiols in type 2 diabetes mellitus: a prospective study. J Clin Transl Endocrinol.

[14] Bourgonje A.R., Gabriëls R.Y., de Borst M.H., Bulthuis M.L.C., Faber K.N., van Goor H. (2019). Serum free thiols are superior to fecal calprotectin in reflecting endoscopic disease activity in inflammatory bowel disease. Antioxidants (Basel).

[15] Boekhoud L., Koeze J., van der Slikke E.C., Bourgonje A.R., Moser J., Zijlstra J.G. (2020). Acute kidney injury is associated with lowered plasma-free thiol levels. Antioxidants (Basel).

[16] Frenay A.S., de Borst M.H., Bachtler M., Tschopp N., Keyzer C.A., van den Berg E. (2016). Serum free sulfhydryl status is associated with patient and graft survival in renal transplant recipients. Free Radic. Biol. Med..

[17] Kao M.P.C., Ang D.S.C., Struthers A.D. (2010). Oxidative stress in renal dysfunction: mechanisms, clinical sequelae and therapeutic options. J. Hum. Hypertens..

[18] Cachofeiro V., Goicochea M., de Vinuesa S.G., Oubiña P., Lahera V., Luño J. (2008). Oxidative stress and inflammation, a link between chronic kidney disease and cardiovascular disease. Kidney Int. Suppl..

[19] Hillege H.L., Janssen W.M.T., Bak A.A.A., Diercks G.F.H., Grobbee D.E., Crijns H.J.G.M. (2001). Microalbuminuria is common, also in a nondiabetic, nonhypertensive population, and an independent indicator of cardiovascular risk factors and cardiovascular morbidity. J. Intern. Med..

[20] Grubb A., Blirup-Jensen S., Lindström V., Schmidt C., Althaus H., Zegers I. (2010). First certified reference material for cystatin C in human serum ERM-DA471/IFCC. Clin. Chem. Lab. Med..

[21] Hu M.L., Louie S., Cross C.E., Motchnik P., Halliwell B. (1993). Antioxidant protection against hypochlorous acid in human plasma. J. Lab. Clin. Med..

[22] Ellman G.L. (1959). Tissue sulfhydryl groups. Arch. Biochem. Biophys..

[23] Inker L.A., Schmid C.H., Tighiouart H., Eckfeldt J.H., Feldman H.I., Greene T. (2012). Estimating glomerular filtration rate from serum creatinine and cystatin C. N. Engl. J. Med..

[24] Pearl J. (2000).

[25] La Bastide-van Gemert S., van den Heuvel E. (2013). Exploring causal hypotheses: breaking with long-standing research traditions. Dev. Med. Child Neurol..

[26] Lederer D.J., Bell S.C., Branson R.D., Chalmers J.D., Marshall R., Maslove D.M. (2019). Control of confounding and reporting of results in causal inference studies. Guidance for authors from editors of respiratory, sleep, and critical care journals. Ann. Am. Thorac. Soc..

[27] Singh-Manoux A., Shipley M.J., Bell J.A., Canonico M., Elbaz A., Kivimäki M. (2017). Association between inflammatory biomarkers and all-cause, cardiovascular and cancer-related mortality. CMAJ (Can. Med. Assoc. J.).

[28] Wonisch W., Falk A., Sundl I., Winklhofer-Roob B.M., Lindschinger M. (2012). Oxidative stress increases continuously with BMI and age with unfavourable profiles in males. Aging Male.

[29] Pencina M.J., D'Agostino R.B., Larson M.G., Massaro J.M., Vasan R.S. (2009). Predicting the 30-year risk of cardiovascular disease: the framingham heart study. Circulation.

[30] Tzoulaki I., Elliott P., Kontis V., Ezzati M. (2016). Worldwide exposures to cardiovascular risk factors and associated health effects: current knowledge and data gaps. Circulation.

[31] Münzel T., Gorit T., Bruno R.M., Taddei S. (2010). Is oxidative stress a therapeutic target in cardiovascular disease?. Eur. Heart J..

[32] Schulz E., Gori T., Münzel T. (2011). Oxidative stress and endothelial dysfunction in hypertension. Hypertens. Res..

[33] Duni A., Liakopoulos V., Roumeliotis S., Peschos D., Dounousi E. (2019). Oxidative stress in the pathogenesis and evolution of chronic kidney disease: untangling ariadne's thread. Int. J. Mol. Sci..

[34] Daenen K., Andries A., Mekahli D., Van Schepdael A., Jouret F., Bammens B. (2019). Oxidative stress in chronic kidney disease. Pediatr. Nephrol..

[35] Oberg B.P., McMenamin E., Lucas F.L., McMonagle E., Morrow J., Ikizler T.A. (2004). Increased prevalence of oxidant stress and inflammation in patients with moderate to severe chronic kidney disease. Kidney Int..

[36] Dounousi E., Papavasiliou E., Makedou A., Ioannou K., Katopodis K.P., Tselepis A. (2006). Oxidative stress is progressively enhanced with advancing stages of CKD. Am. J. Kidney Dis..

[37] Sol M., Kamps J.A.A.M., van den Born J., van den Heuvel M.C., van der Vlag J., Krenning G. (2020). Glomerular endothelial cells as instigators of glomerular sclerotic diseases. Front. Pharmacol..

[38] Li G., Chen Y., Hu H., Liu L., Hu X., Wang J. (2012). Association between age-related decline of kidney function and plasma malondialdehyde. Rejuvenation Res..

[39] Santolini J., Wootton S.A., Jackson A.A., Feelisch M. (2019). The Redox architecture of physiological function. Curr. Opin. Physiol..

[40] Hortin G.L., Sviridov D., Anderson N.L. (2008). High-abundance polypeptides of the human plasma proteome comprising the top 4 logs of polypeptide abundance. Clin. Chem..

[41] Sutton T.R., Minion M., Barbarino F., Koster G., Fernandez B.O., Cumpstey A.F. (2018). A robust and versatile mass spectrometry platform for comprehensive assessment of the thiol redox metabolome. Redox Biol.

[42] Abdulle A.E., van Roon A.M., Smit A.J., Pasch A., van Meurs M., Bootsma H. (2019). Rapid free thiol rebound is a physiological response following cold-induced vasoconstriction in healthy humans, primary Raynaud and systemic sclerosis. Phys. Rep..

[43] McKenna H.T., O'Brien K.A., Fernandez B.O., Minnion M., Tod A., McNally B.D. (2021). Divergent trajectories of cellular bioenergetics, intermediary metabolism and systemic redox status in survivors and non-survivors of critical illness. Redox Biol.

[44] Bourgonje A.R., von Martels J.Z.H., Bulthuis M.L.C., van Londen M., Faber K.N., Dijkstra G. (2019). Crohn's disease in clinical remission is marked by systemic oxidative stress. Front. Physiol..

[45] Anraku M., Chuang V.T., Maruyama T., Otagiri M. (2013). Redox properties of serum albumin. Biochim. Biophys. Acta.

[46] Mullens W., Damman K., Testani J.M., Martens P., Mueller C., Lassus J. (2020). Evaluation of kidney function throughout the heart failure trajectory - a position statement from the Heart Failure Association of the European Society of Cardiology. Eur. J. Heart Fail..

[47] Perrone R.D., Madias N.E., Levey A.S. (1992). Serum creatinine as an index of renal function: new insights into old concepts. Clin. Chem..

[48] van Timmeren M.M., van den Heuvel M.C., Bailly V., Bakker S.J., van Goor H., Stegeman C.A. (2007). Tubular kidney injury molecule-1 in human renal disease. J. Pathol..

[49] Gregg L.P., Tio M.C., Li X., Adams-Huet B., de Lemos J.A., Hedayati S.S. (2018). Association of monocyte chemoattractant protein-1 with death and atherosclerotic events in chronic kidney disease. Am. J. Nephrol..

[50] Atkuri K.R., Mantovani J.J., Herzenberg L.A., Herzenberg L.A. (2007). N-Acetylcysteine--a safe antidote for cysteine/glutathione deficiency. Curr. Opin. Pharmacol..

[51] Deneke S.M. (2000). Thiol-based antioxidants. Curr. Top. Cell. Regul..

[52] Bourgonje A.R., Offringa A.K., van Eijk L.E., Abdulle A.E., Hillebrands J.L., van der Voort P.H.J. (2021). N-acetylcysteine and hydrogen sulfide in coronavirus disease 2019. Antioxid. Redox Signal.

[53] Cumpstey AF, Clark AD, Santolini J, Jackson AA, Feelisch M (2021). COVID-19: a redox disease – what a stress pandemic can teach us about resilience and what we may learn from the reactive species interactome about its treatment. Antioxid. Redox Signal..

