# Supplementary material for: Serum free sulfhydryl status associates with new-onset chronic kidney disease in the general population
Source: Redox Biol. 2021 Dec 9;48:102211. doi: 10.1016/j.redox.2021.102211 (PMC8671125; doi:10.1016/j.redox.2021.102211)
Supplement: Multimedia component 1 [file mmc1.docx]

**Supplementary Tables**

**Supplementary Table S1**. Baseline demographic and clinical characteristics of the study population versus participants excluded because of missing data on protein-adjusted serum free thiols.

|  | **Study population** | **Excluded participants** | ***P*-value** |
| --- | --- | --- | --- |
|  | *n* = 4,745 | *n* = 730 |  |
| **Demographics** |  |  |  |
| Age (years) | 51.4 [42.3-58.8] | 57.4 [48.8-67.0] | <0.001 |
| Male/female | 46.2%/53.8% | 50.4%/49.6% | NS |
| **Anthropometrics** |  |  |  |
| BMI (kg/m^2^) | 25.7 [23.4-28.5] | 27.2 [24.7-30.2] | <0.001 |
| **Risk factors** |  |  |  |
| SBP (mmHg) | 121 [111-113] | 126 [111-133] | <0.001 |
| DBP (mmHg) | 72 [66-78] | 73 [67-79] | NS |
| Smoking: never/former/current | 31.3%/40.8%/27.9% | 26.9%/44.6%/28.5% | NS |
| **Kidney function parameters** |  |  |  |
| eGFR (mL/min/1.73m2) | 96 [85-106] | 92 [82-100] | <0.001 |
| UAE (mg/L) | 7.7 [5.8-11.3] | 8.5 [6.1-14.2] | <0.001 |
| Urine creatinine (mmol/24-h) | 11.9 [9.9-14.5] | 11.6 [9.7-13.9] | <0.05 |

Abbreviations: BMI, body-mass index; DBP, diastolic blood pressure; eGFR, estimated glomerular filtration rate; NS, non-significant; SBP, systolic blood pressure; UAE, urinary albumin excretion.

**Supplementary Table S2**. Stratified analyses for the association between protein-adjusted serum free thiols and the risk of incident CKD across various subgroups.

| **Variable** | **Total (*n*)** | **New-onset CKD (*n*)** | **HR (95% CI)** | ***P*-value for interaction** |
| --- | --- | --- | --- | --- |
| **Overall** | 4745 | 482 | 0.67 (0.47-0.94) |  |
| **Gender** |  |  |  |  |
| Male | 2194 | 274 | 0.66 (0.40-1.08) | 0.632 |
| Female | 2551 | 208 | 0.63 (0.38-1.03) |  |
| **BMI** |  |  |  |  |
| < 25 kg/m^2^ | 2006 | 165 | 0.93 (0.49-1.80) | 0.626 |
| ≥ 25 kg/m^2^ | 2739 | 317 | 0.60 (0.40-0.91) |  |
| **Hypertension** |  |  |  |  |
| No | 3536 | 270 | 0.59 (0.36-0.97) | **<0.001** |
| Yes | 1207 | 211 | 0.77 (0.48-1.25) |  |
| **CHF** |  |  |  |  |
| No | 4653 | 463 | 0.73 (0.51-1.04) | **0.024** |
| Yes | 89 | 19 | 0.15 (0.02-0.91) |  |
| **History of CVD** |  |  |  |  |
| No | 4620 | 460 | 0.68 (0.48-0.97) | 0.335 |
| Yes | 125 | 22 | 0.19 (0.02-1.61) |  |
| **UAE** |  |  |  |  |
| < 7.7 mg/24-h | 2372 | 101 | 0.36 (0.18-0.69) | **<0.001** |
| > 7.7 mg/24-h | 2373 | 381 | 0.80 (0.54-1.20) |  |
| **eGFR** |  |  |  |  |
| < 95 mL/min/1.73m^2^ | 2372 | 312 | 0.66 (0.43-1.00) | 0.961 |
| > 95 mL/min/1.73m^2^ | 2372 | 170 | 0.69 (0.37-1.28) |  |
| **Current smoking** |  |  |  |  |
| No | 3380 | 340 | 0.76 (0.50-1.15) | 0.376 |
| Yes | 1306 | 134 | 0.49 (0.26-0.92) |  |
| **Total cholesterol** |  |  |  |  |
| < 5.36 mmol/L | 2353 | 217 | 0.66 (0.40-1.11) | 0.115 |
| > 5.36 mmol/L | 2370 | 264 | 0.69 (0.43-1.10) |  |
| **Triglycerides** |  |  |  |  |
| < 1.06 mmol/L | 2322 | 203 | 0.84 (0.50-1.41) | 0.442 |
| > 1.06 mmol/L | 2317 | 263 | 0.55 (0.33-0.90) |  |

Abbreviations: CKD, chronic kidney disease; CI, confidence interval; HR, hazard ratio; eGFR, estimated glomerular filtration rate; UAE, urinary albumin excretion; BMI, body-mass index; CHF, chronic heart failure.
